# Gender-Specific Associations between Circulating T-Cadherin and High Molecular Weight-Adiponectin in Patients with Stable Coronary Artery Disease

**DOI:** 10.1371/journal.pone.0131140

**Published:** 2015-06-17

**Authors:** Andreas W. Schoenenberger, Dennis Pfaff, Boris Dasen, Agne Frismantiene, Paul Erne, Therese J. Resink, Maria Philippova

**Affiliations:** 1 Division of Geriatrics, Department of General Internal Medicine, Inselspital, Bern University Hospital and University of Bern, Bern, Switzerland; 2 Department of Biomedicine, Laboratory for Signal Transduction, University Hospital Basel and University of Basel, Basel, Switzerland; University of Louisville, UNITED STATES

## Abstract

Close relationships exist between presence of adiponectin (APN) within vascular tissue and expression of T-cadherin (T-cad) on vascular cells. APN and T-cad are also present in the circulation but here their relationships are unknown. This study investigates associations between circulating levels of high molecular weight APN (HMW-APN) and T-cad in a population comprising 66 women and 181 men with angiographically proven stable coronary artery disease (CAD). Plasma HMW-APN and T-cad were measured by ELISA and analysed for associations with baseline clinical characteristics and with each other. In multivariable analysis BMI and HDL were independently associated with HMW-APN in both genders, while diabetes and extent of coronary stenosis were independently associated with T-cad in males only. Regression analysis showed no significant association between HMW-APN and T-cad in the overall study population. However, there was a negative association between HMW-APN and T-cad (*P*=0.037) in a subgroup of young men (age <60 years, had no diabetes and no or 1-vessel CAD) which persisted after multivariable analysis with adjustment for all potentially influential variables (*P*=0.021). In the corresponding subgroup of women there was a positive association between HMW-APN and T-cad (*P*=0.013) which disappeared after adjustment for HDL. After exclusion of the young men, a positive association (*P*=0.008) between HMW-APN and T-cad was found for the remaining participants of the overall population which disappeared after adjustment for HDL and BMI. The existence of opposing correlations between circulating HMW-APN and T-cad in male and female patient populations underscores the necessity to consider gender as a confounding variable when evaluating biomarker potentials of APN and T-cad.

## Introduction

Adiponectin (APN), a 28-30kDa protein abundantly secreted into the circulation by adipocytes, possesses anti-inflammatory, anti-atherogenic, insulin-sensitizing and cardioprotective properties (reviewed in [1–4]). Circulating APN exists in different forms (monomeric, oligomeric and multimeric, high-molecular-weight (HMW)) [1–4], and among them the HMW oligomer is most abundant and considered physiologically most relevant [1–4]. Plasma concentrations of APN have been frequently found to be reduced in obesity, metabolic syndrome, type 2 diabetes, hypertension, coronary artery disease (CAD) and acute coronary syndrome (ACS) [1,2].

T-cadherin (T-cad), a cell surface glycosylphosphatidylinositol (GPI)-anchored cadherin family member, is a binding protein for hexameric and HMW-APN [5]. In vascular tissue T-cad and APN display common cellular localization patterns. T-cad is expressed on endothelial cells (EC) and vascular smooth muscle cells (SMC) with high expression in the subendothelial SMC layer [6]. APN is present on the luminal surface of human atherosclerotic tissues [7] and murine aorta [8,9], and also localizes to the subendothelial and medial layers of catheter-injured rat aortae [10] and human atherosclerotic lesions with damaged endothelium [7]. Studies using T-cad-deficient mice have demonstrated that T-cad expression on EC is essential both for binding and localization of APN to vascular tissues [8,9,11] and for mediating ameliorative revascularization actions of APN following cardiac ischemia-reperfusion injury and hind limb ischemia [11,12].

In addition to its cellular localization, T-cad is also present in the circulation as a component of microparticles (MPs) [13], which are submicron plasma membrane vesicles shed into the extracellular space from cells undergoing stress, activation or apoptosis [14]. MPs are now widely considered as both biomarkers and active participants (beneficial and/or detrimental) for numerous diseases with vascular involvement [14]. *In vitro* and *in vivo* studies have shown that T-cad is shed from stressed/apoptotic EC and in amounts reflecting the extent of EC activation and damage [13]. Furthermore, and similarly to the vasculoprotective actions of T-cad expressed on the EC surface, MP-conveyed T-cad induces prosurvival signal transduction and angiogenic behaviour in target EC [13,15–18]. These actions of T-cad are independent of its function as a receptor for APN and are mediated *via* homophilic ligation (T-cad-T-cad) interactions [13,15–18]. Given the importance of EC surface-expressed T-cad for recruitment of APN to vascular tissues [8,9,11], shedding of T-cad from the surface of EC into the circulation might possibly also influence circulating levels of APN.

Associations between levels of APN and T-cad in the circulation have never been studied. However, information regarding biomarker relevance for circulating T-cad does suggest some analogies with circulating APN, for which hypoadiponectemia is typically found in diabetes, metabolic syndrome and coronary artery disease [1,2]. Plasma concentrations of T-cad were found to be decreased in association with increasing severity of coronary artery disease and a higher risk for ACS [19]. Further, in the overall population (comprising patients with normal coronary arteries, chronic CAD or ACS) levels of circulating T-cad were lower in males, in patients with diabetes or hypertension, negatively correlated with body mass index (BMI) and positively correlated with high density lipoprotein (HDL) [19]. These observations, taken together with all experimental evidence for common expression patterns for APN and T-cad within vascular tissue, their direct physical interaction and their involvement in similar pathophysiological processes led us to hypothesize that there might be some relationships/correlations between the levels of APN and T-cad in the circulation. In order to test this hypothesis we performed a parallel analysis of HMW-APN and T-cad in plasma from patients with stable CAD and evaluated their individual associations with baseline clinical characteristics and their associations with each other.

## Patients and Methods

### Study population

The subjects include patients who underwent coronary angiography for the evaluation of CAD at the hospital of Lucerne, Switzerland. The decision to perform coronary angiography was made by the cardiologist in charge based on non-invasive clinical examinations. We excluded patients who presented acutely with ST-segment elevation myocardial infarction, non-ST-segment elevation myocardial infarction or unstable angina, because the acute event can perturb APN and T-cad [19,20]. The present study therefore embraced patients with stable condition. All patients had either CAD (defined as coronary stenosis of 50% or more in at least 1 coronary vessel) or coronary sclerosis (defined as angiographically visible coronary irregularities of less than 50% lumen narrowing). All patients provided written informed consent. The institutional ethical committee approved the study (Ethikkommission des Kantons Luzern, approval no. 536), which was conducted in compliance with the Declaration of Helsinki.

### Clinical measurements

In all patients, clinical characteristics (age, gender, cardiovascular risk factors, medical history, and clinical presentation) were assessed at baseline. Hypertension was defined as increased blood pressure (BP) ≥140/90 mmHg, or current treatment for hypertension. Dyslipidemia was defined as total cholesterol >234 mg/dl (6.0 mmol/L) or low-density lipoprotein cholesterol >117 mg/dl (3.0 mmol/L), or usage of drug therapies for dyslipidemia. Diagnosis of diabetes mellitus was made if fasting plasma glucose was ≥7 mmol/L on ≥2 different days or if postprandial plasma glucose was ≥11.1 mmol/L. Patients were considered smokers if they currently smoked ≥1 cigarette per week. Patients who previously stopped smoking were considered nonsmokers. A positive family history of CAD was defined as evidence of CAD in a parent or sibling <60 years old. We defined metabolic syndrome as BMI ≥30 kg/m^2^ and the presence of at least two of either hypertension, dyslipidemia or diabetes [21].

### Measurement of T-cad and HMW-APN in plasma

Blood samples were drawn after overnight fasting from all patients prior to elective angiography after a 20–30 min resting period in the supine position. Blood samples drawn into 10-ml sodium heparin vacutainer tubes (BD Biosciences, Erembodegem, Belgium) were centrifuged at 3’500x*g* for 25 min at room temperature. The cell-free plasma samples were aliquoted into polypropylene tubes, snap-frozen and stored at—70°C until analysis. The concentration of T-cad in plasma was determined by double sandwich immunoassay on the Meso Scale Discovery electrochemiluminescence platform (MSD; Rockville, Maryland, USA) following protocols exactly as detailed previously [19]. Capture antibody was polyclonal mouse anti-T-cadherin antibody (Sigma-Aldrich Chemie, Buchs, Switzerland) and detection was performed using biotin-conjugated goat polyclonal anti-T-cadherin antibody (R&D Systems Europe Ltd., Abingdon, UK) and streptavidin Sulfo-TAG (MSD). The concentration of HMW-APN in plasma was measured by enzyme-linked immunosorbent assay (ELISA) using the Quantikine ELISA kit for human HMW Adiponectin/Acrp30 (R&D Systems Europe Ltd., Abingdon, UK). The assays were run according to the manufacturer’s protocol. Concentrations of T-cad and HMW-APN in plasma samples were measured blinded to the clinical data.

### Statistical Analysis

First, we excluded patients from the analysis who had HMW-APN/T-cad-ratio ≥200, based on the assumption that these patients probably had measurement error of HMW-APN and/or T-cad. Second, we imputed missing values for cholesterol measurements in 5 patients using the median values. Third, we descriptively analyzed baseline findings for the overall study population. Baseline characteristics were also analyzed separate for male and female participants based on our observations during preliminary analyses that there were gender-specific differences in HMW-APN and T-cad. Fourth, we used linear regression models to search for bivariable associations between HMW-APN or T-cad and eighteen baseline findings separate for male and female participants. We searched for associations with all investigated baseline characteristics, but only report those that showed a significant association. For each regression model, R^2^, coefficients and p values are provided. Owing to skewness, HMW-APN was log transformed for linear regression analysis using the natural logarithm. Fifth, we used a multivariable linear regression model to determine independent associations of baseline findings with HMW-APN or T-cad as dependent variables. As independent variables, we selected all baseline variables that showed significant associations in bivariable analysis. Sixth, we performed bivariable linear regression analysis to search for associations between log transformed HMW-APN and T-cad. For significant associations, multivariable models were additionally performed with adjustment for each of the following variables: age, sex, BMI, blood pressure, hypertension, dyslipidemia, diabetes, CAD severity, HDL and triglycerides. These analyses were performed in the overall population, separate for both genders, and according to age (age < 60 years *vs*. age ≥ 60 years). We used Student's *t* test to compare continuous variables with normal distribution and Wilcoxon rank-sum test for variables with non-normal distribution. For categorical variables, chi-square test or, if cell counts were less than 5, Fisher’s exact test was used. Data were analyzed using STATA 12.1 (StataCorp, College Station, Texas, USA). A significance level of 0.05 was assumed for all tests.

## Results

### Study group characteristics

The study population embraced 250 patients. Three patients (1.2%) with probable measurement error for HMW-APN and/or T-cad were excluded resulting in an analysed population of 247 patients. Table 1 presents the demographic and clinical characteristics of the study group as a whole and according to gender. Male study participants had higher BMI, lower HDL cholesterol and higher creatinine than female study participants, they more often were on a prescription for ACE inhibitors, ARBs or statins, and they had more severe CAD on angiography.

**Table 1:** Demographic and clinical characteristics of the study group and according to gender.

Variable	All patients (n = 247)	Male (n = 181)	Female (n = 66)	*P* value
Age (years), mean ± SD	60.7 ± 9.9	60.2 ± 10.0	62.2 ± 9.5	0.174
BMI (kg/m^2^), mean ± SD	27.4 ± 4.7	27.8 ± 4.5	26.3 ± 4.9	0.011
Systolic BP (mmHg), mean ± SD	131 ± 18	130 ± 18	133 ± 20	0.403
Diastolic BP (mmHg), mean ± SD	75 ± 10	76 ± 10	73 ± 11	0.108
***Cardiovascular risk factors***				
Hypertension, n (%)	161 (65.2)	117 (64.6)	44 (66.7)	0.767
Diabetes, n (%)	42 (17.0)	32 (17.7)	10 (15.2)	0.640
Current smoker, n (%)	52 (21.1)	42 (23.2)	10 (15.2)	0.170
Dyslipidemia, n (%)	180 (72.9)	136 (75.1)	44 (66.7)	0.185
Family history of CAD, n (%)	95 (38.5)	64 (35.4)	31 (47.0)	0.097
Metabolic syndrome, n (%)	44 (17.8)	35 (19.3)	9 (13.6)	0.300
***Medication*** ^***1***^				
ACE inhibitor/ARB, n (%)	108 (43.7)	86 (47.5)	22 (33.3)	0.047
Betablocker, n (%)	137 (55.5)	103 (56.9)	34 (51.5)	0.451
Statin, n (%)	147 (59.5)	114 (63.0)	33 (50.0)	0.066
***Medical history and co-morbidities***				
Previously known CAD, n (%)	54 (21.9)	45 (24.9)	9 (13.6)	0.059
History of ACS, n (%)	13 (5.3)	11 (6.1)	2 (3.0)	0.523
History of stroke, n (%)	11 (4.5)	10 (5.5)	1 (1.5)	0.297
Known PAD, n (%)	18 (7.3)	13 (7.2)	5 (7.6)	0.916
Known COPD, n (%)	10 (4.1)	7 (3.9)	3 (4.6)	0.730
***Findings during coronary angiography***				
Number of coronary arteries with stenosis ≥50%, n (%)				
→ 0	55 (22.3)	34 (18.8)	21 (31.8)	0.011
→ 1	73 (29.6)	49 (27.1)	24 (36.4)	
→ 2	60 (24.3)	47 (26.0)	13 (19.7)	
→ 3	59 (23.9)	51 (28.2)	8 (12.1)	
***Lipid measurements and creatinine***				
LDL cholesterol (mmol/L), mean ± SD	2.7 ± 1.1	2.6 ± 1.1	2.9 ± 1.1	0.055
HDL cholesterol (mmol/L), mean ± SD	1.4 ± 0.4	1.3 ± 0.4	1.7 ± 0.4	<0.001
Triglycerides (mmol/L), mean ± SD	1.5 ± 1.1	1.5 ± 1.2	1.3 ± 0.7	0.635
Creatinine (μmol/L), mean ± SD	80 ± 16	83 ± 15	71 ± 14	<0.001
***HMW-adiponectin and T-cadherin***				
HMW-adiponectin (μg/mL), mean ± SD	5.5 ± 3.8	4.6 ± 3.1	7.9 ± 4.4	<0.001
T-cadherin (ng/mL), mean ± SD	143.7 ± 33.2	141.7 ± 32.5	149.2 ± 34.7	0.116

Abbreviations: ACE, angiotensin-converting enzyme; ACS, acute coronary syndrome; ARB, angiotensin receptor blocker; BP, blood pressure; CAD, coronary artery disease; COPD, chronic obstructive pulmonary disease; HDL, high-density lipoprotein; LDL, low-density lipoprotein; PAD, peripheral artery disease; SD, standard deviation. P values compare female and male study participants.

^1^ Long-term medication within two weeks before study inclusion.

Table 1 also displays HMW-APN and T-cad values: female study participants had significantly higher HMW-APN than male participants (as expected [1–4]), whereas for T-cad only a statistically non-significant trend towards higher values in females was found.

### Associations of HMW-APN and T-cad with baseline characteristics

Table 2 shows bivariable associations of circulating HMW-APN and T-cad with baseline characteristics separate for male and female participants. Plasma levels of HMW-APN correlated negatively with BMI and positively with HDL in both males and females. As measured by the R^2^, these associations were relatively strong. For both genders, negative correlations were found between HMW-APN and certain cardiovascular risk factors (dyslipidemia in males; systolic BP and diabetes in females). In addition, HMW-APN positively correlated with age and negatively with previously known CAD and triglycerides in males. Plasma levels of T-cad correlated negatively with BMI, the presence of hypertension and the use of an ACE inhibitor or ARB in both males and females. Further negative associations were found with diabetes and the extent of CAD in males, and with systolic BP in females. In female participants, T-cad positively correlated with HDL.

**Table 2:** Bivariable associations between circulating HMW-adiponectin or T-cadherin and baseline characteristics according to gender.

Characteristic	HMW-adiponectin						T-cadherin					
	*Male*			*Female*			*Male*			*Female*		
	*R* ^*2*^	*Coeff*	*P value*	*R* ^*2*^	*Coeff*	*P value*	*R* ^*2*^	*Coeff*	*P value*	*R* ^*2*^	*Coeff*	*P value*
Age	0.04	0.01	0.009	0.02	0.01	0.206	0.01	-0.31	0.199	0.01	-0.27	0.556
BMI	0.08	-0.04	<0.001	0.19	-0.05	<0.001	0.03	-1.24	0.020	0.06	-1.75	0.046
Systolic BP	0.00	0.00	0.459	0.14	-0.01	0.002	0.02	-0.25	0.066	0.09	-0.53	0.016
Hypertension	0.00	-0.06	0.531	0.04	-0.25	0.101	0.05	-14.9	0.003	0.06	-18.3	0.042
Diabetes	0.00	0.03	0.815	0.13	-0.57	0.003	0.06	-20.2	0.001	0.00	-4.6	0.705
Dyslipidemia	0.04	-0.31	0.005	0.00	-0.03	0.858	0.00	3.52	0.530	0.00	-2.43	0.791
ACE inhibitor/ARB	0.00	0.06	0.557	0.03	-0.21	0.158	0.03	-11.5	0.017	0.06	-18.2	0.044
Number of coronary arteries with stenosis ≥50%	0.01	-0.04	0.327	0.00	0.01	0.872	0.03	-5.38	0.016	0.01	-2.95	0.498
HDL cholesterol	0.14	0.66	<0.001	0.14	0.48	0.002	0.00	2.21	0.737	0.07	21.2	0.027
Triglycerides	0.07	-0.15	<0.001	0.03	-0.14	0.152	0.00	-1.74	0.408	0.04	-9.48	0.107

Abbreviations: ACE, angiotensin-converting enzyme; ARB, angiotensin receptor blocker; BMI, body mass index; BP, blood pressure; CAD, coronary artery disease; CI, confidence interval; HDL, high-density lipoprotein.

Multivariable analyses showed that the negative association of BMI with HMW-APN was independent from other baseline variables in males (coefficient -0.02 [95% CI -0.04 –-0.00], *P* = 0.045) and females (coefficient -0.03 [95% CI -0.06 –-0.00], *P* = 0.038). Using multivariable analyses, the positive association of HDL with HMW-APN was independent from other baseline variables in males (coefficient 0.43 [95% CI 0.16–0.71], *P* = 0.002) and nearly independent in females (coefficient 0.23 [95% CI -0.07–0.54], *P* = 0.129). Other significant bivariable associations between baseline variables and HMW-APN were weaker in multivariable analyses. Regarding T-cad, presence of diabetes (coefficient -14.77 [95% CI -27.12 –-2.43], *P* = 0.019) and numbers of coronary vessels with significant stenosis (coefficient -4.35 [95% CI -8.62 –-0.09], *P* = 0.045) were negatively and independently associated in males, whereas other baseline findings in males and findings in females showed no association in multivariable analyses.

### Associations between levels of HMW-APN and T-cad

**Fig 1:**
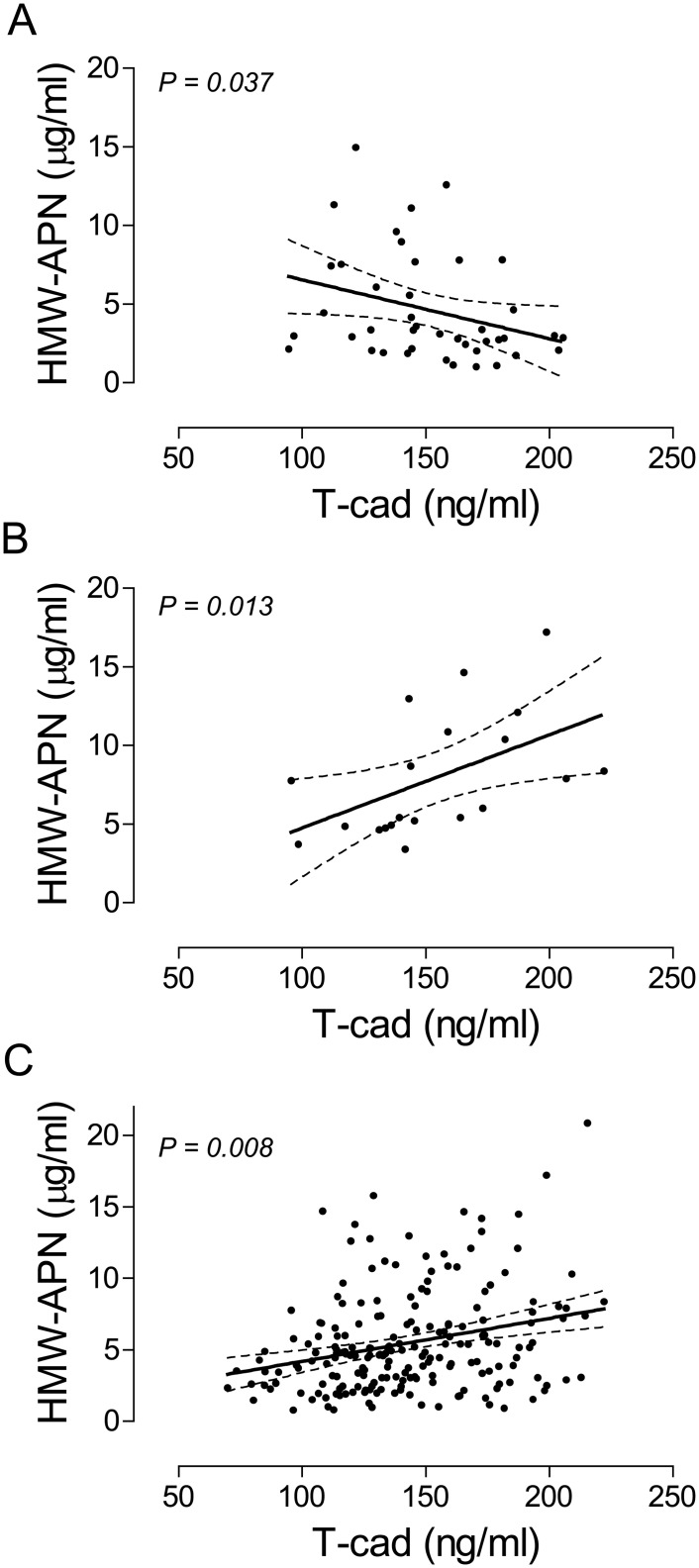
Regression plots showing relationships between HMW-APN and T-cad. Associations between HMW-APN and T-cad in the subgroups of young (age < 60, had no diabetes and no or 1-vessel CAD) males (A) or females (B) and in the overall population minus the young male subgroup (C) were determined by bivariable linear regression analyses. Data are presented as fitted regression plots (solid lines) with 95% confidence intervals (dashed lines) for the fits and significance levels (*P*).

In the overall study population, no significant association was found between HMW-APN and T-cad (R^2^ 0.01, coefficient 0.002 [95% CI -0.001–0.004], *P* = 0.144). However, we observed a significant negative association in a subgroup of 42 younger men (age < 60 years) who were relatively healthy (had no diabetes and no or 1-vessel CAD) (R^2^ 0.10, coefficient -0.008 [95% CI -0.015 –-0.001], *P* = 0.037) (Fig 1A). The significant association persisted in the multivariable model (*P* = 0.021). In contrast, in the corresponding subgroup of 20 young and healthy women a relatively strong positive association was found (R^2^ 0.30, coefficient 0.008 [95% CI 0.002–0.013], *P* = 0.013) (Fig 1B). In this group, the significant association persisted for all adjustment variables with the exception of HDL, where there was no longer an association between HMW-APN and T-cad (*P* = 0.592). After exclusion of the 42 young and healthy men, a significant positive association between HMW-APN and T-cad was found for the 205 remaining participants of the overall population (R^2^ 0.03, coefficient 0.004 [95% CI 0.001–0.006], *P* = 0.008) (Fig 1C). This significant association disappeared after adjustment for BMI (*P* = 0.117) and HDL (*P* = 0.131).

## Discussion

This prospective cohort study of patients with chronic stable CAD is the first to investigate associations between circulating HMW-APN and circulating T-cad. The most significant finding of our study is that associations between HMW-APN and T-cad were gender-specific in patients < 60 years, being positive in females but negative in males. Multivariable regression analysis with adjustment for age, sex, BMI, BP, hypertension, dyslipidemia, diabetes, CAD severity, HDL and triglycerides revealed that HDL is the relevant contributor to the positive association between HMW-APN and T-cad in females, while the negative association in males was independent of all the adjusted variables.

We have reported higher levels of circulating T-cad in females *vs*. males in a cohort embracing patients with normal coronary arteries, stable CAD and ACS [19], whereas in the present study on only patients with stable CAD plasma T-cad was not significantly different between males and females. The gender dimorphism of our previous study may therefore reflect that T-cad levels were lowest in the ACS patient group and that this group consisted predominantly (80%) of males [19]. The present study shows that gender issues are important only for T-cad *per se* but also for associations between T-cad and HMW-APN.

The gender-related association between T-cad and HMW-APN may relate to hormonal control. Both proteins can be regulated by androgens and estrogens. APN secretion by adipocytes is suppressed by testosterone [22,23] and estrogen [24]. Data on the correlations of circulating APN with hormonal status in women are conflicting. Some investigators have shown that transition to menopause is accompanied with increased circulating APN [25], which is consistent with the data on estrogen-dependent suppression of APN [24]. Others, however, have reported lack of correlations between age and APN in females [22,26] or between serum APN and estradiol levels [27,28], very small [25] or no [27] differences in APN between pre- and post-menopausal women, and very small [28] or no [27] effects of estrogen replacement therapy on APN in post-menopausal females. With respect to T-cad, its expression was upregulated in castrated mice and downregulated following androgen replacement [29], while estradiol and progesterone were reported to upregulate T-cad expression in osteosarcoma cells *in vitro* [30]. We did not measure serum levels of sex hormones and therefore cannot asses causative contribution to the gender-specific associations between HMW-APN and T-cad reported herein. Nevertheless, our finding from multivariable analysis that the positive association in younger females depended on HDL may support our suggestion of some hormonal influence on the relationship between T-cad and HMW-APN-APN relationship since women have higher HDL levels due to actions of estrogen [31,32].

Regulation of APN and T-cad by hormones or other effectors is further complicated by the fact that the two proteins reciprocally influence their expression levels. T-cad-deficient mice exhibited elevated levels of circulating HMW-APN [11,12] and genome-wide association studies have linked variants in T-cad gene to elevated [33] or decreased [34] circulating levels of APN in humans, supporting that circulating APN is influenced by the presence and/or functionality of T-cad. Conversely, APN-deficient mice exhibited a reduced tissue level of T-cad protein that was restorable *in vivo* by delivery of exogenous APN [11,12]. Administration of APN to cardiomyocytes injured by hypoxia/reoxygenation *in vitro* also upregulated T-cad protein expression [35], and in a model of liver fibrosis elevated circulating APN was associated with elevated hepatic expression of T-cad [36]. Thus, tissue T-cad can negatively regulate circulating APN levels by promoting its clearance from blood and sequestration to tissues (“negative T-cad-to-APN regulation”) [9,11,12], while APN can positively regulate tissue T-cad expression (“positive APN-to-T-cad feedback regulatory loop”) [9,11]. Such bidirectional regulation may permit us to formulate a working hypothesis that attempts to explain the observed gender-dependent associations between circulating APN and T-cad. In young men who have relatively low serum APN levels (*vs*. older men and especially women) T-cad efficiently sequesters APN from blood (i.e. “negative T-cad-to-APN regulation” is active); however a low level of APN may not be sufficient to induce the “positive APN-to-T-cad feedback regulatory loop”, so the resulting association between two proteins in plasma is negative. On the other hand in women higher serum APN levels result in less efficient APN clearance by T-cad and APN-dependent upregulation of T-cad expression is initiated in order to meet increased needs for APN clearance; in this circumstance the “positive APN-to-T-cad feedback regulatory loop” overrides “negative T-cad-to-APN regulation”, and the resulting correlation between the protein levels in plasma is positive. In an overall population the opposing gender-associated correlations "neutralize" each other or co-exist in a bidirectional, intermediate state of equilibrium, so the resulting analysis shows no correlation between APN and T-cad.

Whatever the mechanisms involved, dynamic modulation of APN and/or T-cad levels may have important implications for the course of cardiovascular disease since both molecules exert anti-atherogenic and revascularization actions. In EC APN exerts a number of vasculoprotective actions that include suppression of adhesion molecules, superoxide generation and apoptosis, and the promotion of angiogenesis/revascularization [2,11,37,38]. Importantly, and although APN-induced signaling in EC is mediated *via* AdipoR1 and AdipoR2, this signalling crucially depends upon the presence of T-cad on EC [11,12,38]. T-cad has thus been attributed functions as a co-receptor that localizes APN to the endothelium for presentation to signal transduction-competent AdipoR1 or AdipoR2 [39]. Independently of this co-receptor function, T-cad *per se* expressed either on the surface of EC or as a component of circulating EC-derived microparticles promotes prosurvival and proangiogenic behaviour in EC [13,15–18]. Reduced tissue (and thereby circulating) T-cad will both diminish its reparative and protective functions in the vasculature [19] and impair binding of APN molecules to the endothelium with a concomitant reduction of APN-driven vasculoprotective signals [11,12]. Further studies aimed at analysis of changes in circulating APN and T-cad levels in parallel with histochemical analyses of the presence, expression level and localization pattern of their cell-bound pools in the vascular wall at different stages of cardiovascular disease will help to better understand the impact of dynamic interactions between the two proteins on the progression on cardiovascular disorders.

It needs to be recognized that changes in circulating T-cad may not exclusively reflect the state of endothelial integrity and/or dysfunction and that the concentration of circulating APN does not solely reflect the balance of sequestration to, and release from, the endothelium. T-cad was first detected (by FACS analysis) in human plasma as a component of EC-derived microparticles [13] and thus its concentration in the circulation was presumed to reflect a combination of the burden of protein expressed on the surface of EC, extent of endothelial activation and MP shedding and degree of endothelial erosion [19]. However, since plasma levels of T-cad were analysed by ELISA we cannot exclude contributions from other sources or forms (e.g. cleaved or secreted) of T-cad. SMC abundantly express T-cad and can also shed MPs [40]. T-cad is also expressed in adipose tissue [41] and was very recently identified (by ESI-LC-MS/MS proteomic profiling) as a novel component of the secretome of adipocytes isolated from murine visceral adipose tissue [42]. Moreover, levels of T-cad were much less abundant in the secretome from diabetes-susceptible mice [42], which is concordant with the previous finding of lower levels of T-cad in diabetic patients as compared to patients without diabetes [19]. Circulating APN is predominantly derived from adipocytes but several other cell types including cardiomyocytes [43], SMC [44] and EC [8,45] can also synthesize and secrete APN. T-cad is expressed on the surface of these cell types [46–48] and therefore locally produced APN together with circulating adipocyte-derived APN may have important autocrine mechanisms of action.

### Study limitations

First, the study was performed at a single center which may preclude generalizability. Second, the study is limited by reliance on data obtained from a single blood collection from each patient. Third, we measured only HMW-APN and not total APN. However very close correlations between total APN and HMW-APN (*r* ^*2*^ = 0.969, *P* < 0.0001 [49] and *r* ^*2*^ = 0.786, *P* < 0.0001 [50]) together with effective biomarker usefulness of HMW-APN (*vs*. total APN or HMW-total ratio) [49–51] have been demonstrated, and in the context of cardiovascular protective actions HMW-APN is regarded as the active form of this adipokine [49,51,52]. Additionally, in the context of associations between T-cad and APN a number of genome-wide studies revealed that association of SNPs in *CDH13* gene were much stronger with levels of HMW-APN as compared total APN levels [33,53]. Fourth, and with regard to gender-issues, levels of testosterone and estradiol were not measured.

### Conclusions

This study in patients with stable CAD demonstrates the existence of gender-specific associations for circulating HMW-APN and T-cad with baseline clinical characteristics and important opposing correlations between circulating APN and T-cad in male and female patients aged < 60 years. The findings are indicative of a complexity of mechanisms underlying the APN and/or T-cad regulated axis of cardiovascular disease and underscore the necessity to consider gender as a confounding variable when evaluating biomarker potentials of APN and T-cad. Larger prospective studies in populations across the cardiovascular disease spectrum are warranted, in particular studies that elucidate the causal relationship between these biomarkers.
